# A Novel Large In-Frame Deletion within the *CACNA1F* Gene Associates with a Cone-Rod Dystrophy 3-Like Phenotype

**DOI:** 10.1371/journal.pone.0076414

**Published:** 2013-10-04

**Authors:** Jan Hauke, Andrea Schild, Antje Neugebauer, Alexandra Lappa, Julia Fricke, Sascha Fauser, Stefanie Rösler, Andrea Pannes, Dirk Zarrinnam, Janine Altmüller, Susanne Motameny, Gudrun Nürnberg, Peter Nürnberg, Eric Hahnen, Bodo B. Beck

**Affiliations:** 1 Institute of Human Genetics, University of Cologne, Cologne, Germany; 2 Center for Molecular Medicine Cologne (CMMC), University of Cologne, Cologne, Germany; 3 Center of Familial Breast and Ovarian Cancer, University Hospital of Cologne, Cologne, Germany; 4 Department of Ophthalmology, University of Cologne, Cologne, Germany; 5 Medical Practice Dr. Zarrinnam, Heinsberg, Germany; 6 Cologne Center for Genomics, University of Cologne, Cologne, Germany; 7 Cologne Excellence Cluster on Cellular Stress Responses in Aging-Associated Diseases (CECAD), University of Cologne, Cologne, Germany; The University of Melbourne, Australia

## Abstract

Cone-rod dystrophies (CORDs) represent a heterogeneous group of monogenic diseases leading to early impairment of vision. The majority of CORD entities show autosomal modes of inheritance and X-linked traits are comparably rare. So far, three X-chromosomal entities were reported (CORDX1, -X2 and -X3). In this study, we analysed a large family of German origin with solely affected males over three generations showing a CORDX-like phenotype. Due to the heterogeneity of cone-rod dystrophies, we performed a combined linkage and X-exome sequencing approach and identified a novel large intragenic in-frame deletion encompassing exons 18 to 26 within the *CACNA1F* gene. *CACNA1F* is described causative for CORDX3 in a single family originating from Finland and alterations in this gene have not yet been reported in other CORDX pedigrees. Our data independently confirm *CACNA1F* as the causative gene for CORDX3-like phenotypes and detailed clinical characterization of the family expands the knowledge about the phenotypic spectrum of deleterious *CACNA1F* alterations.

## Introduction

Cone photoreceptor cells in the human retina are responsible for daylight vision, high visual acuity and colour discrimination. Thus, inherited human diseases primarily affecting the cone system lead to severe visual impairment. Patients with these disorders mostly experience first symptoms early in life and usually show a decreased visual acuity, and variable degrees of colour vision defects, photophobia and nystagmus. According to the course of the disease, disorders of cone function can be divided into stationary (cone dysfunction syndromes) and progressive disorders (cone or cone—rod dystrophies). However, there is remarkable inter- and intrafamilial variation with respect to age of onset and severity of symptoms [1-4]. While both cone dystrophies (CODs) and cone-rod dystrophies (CORDs) share primary cone degeneration as common feature, CORDs additionally show a peripheral retinal involvement and the electroretinogram (ERG) is characterized by a decrease in both cone and rod responses, with cone responses more severely affected than rod-specific ERG components [5]. In later CORD stages, advanced impairment of rod function frequently results in night blindness when the rod system also becomes affected.

Genetically, non-syndromic CODs and CORDs represent a heterogeneous group of monogenic diseases with more than 20 causative genes identified [5]. While the vast majority of CODs/CORDs show autosomal modes of inheritance, X-linked traits are comparably rare [6]. So far, three X-chromosomal recessive CORD entities were reported: CORDX1 (MIM 304020; Xp11.4) results from mutations in the alternatively spliced open reading frame 15 variant (ORF15) of the retinitis pigmentosa G-coupled receptor (RPGR) gene [7] and is regarded as the most common X-linked CORD [5,8-11]. CORDX2 (MIM 300085) has been mapped to the long arm of the X chromosome (Xq27.2-28) in a single family while the underlying genetic aberration remains to be identified [12]. CORDX3 (MIM 300476) was mapped in a single family originating from Finland [11,13,14] and subsequent studies revealed a splicing mutation within the *CACNA1F* gene encoding the voltage-dependent calcium channel alpha 1F subunit [15]. To our knowledge, mutations of the *CACNA1F* gene have not been confirmed in independent CORDX pedigrees. Notably, damaging alterations of the *CACNA1F* gene are mostly associated with incomplete X-linked congenital stationary night blindness type 2A (CSNB2A, MIM 300071), a non-progressive retinal disorder [1,2,16]. In this study, we report the analysis of a large family of German origin with solely affected males over three generations showing a CORDX-like phenotype. Due to the heterogeneity of cone rod dystrophies and the additional characteristics present in this family, we performed a combined linkage and X-exome sequencing approach in order to identify the underlying mutation. Using pooled patient DNA samples, we identified a novel intragenic in-frame deletion within *CACNA1F*. Our data independently confirm *CACNA1F* as the causative gene for CORDX3-like phenotypes and detailed clinical characterization of the family expands the knowledge about the phenotypic spectrum of *CACNA1F* mutations.

## Materials and Methods

### Subjects and DNA isolation

**Figure 1:**
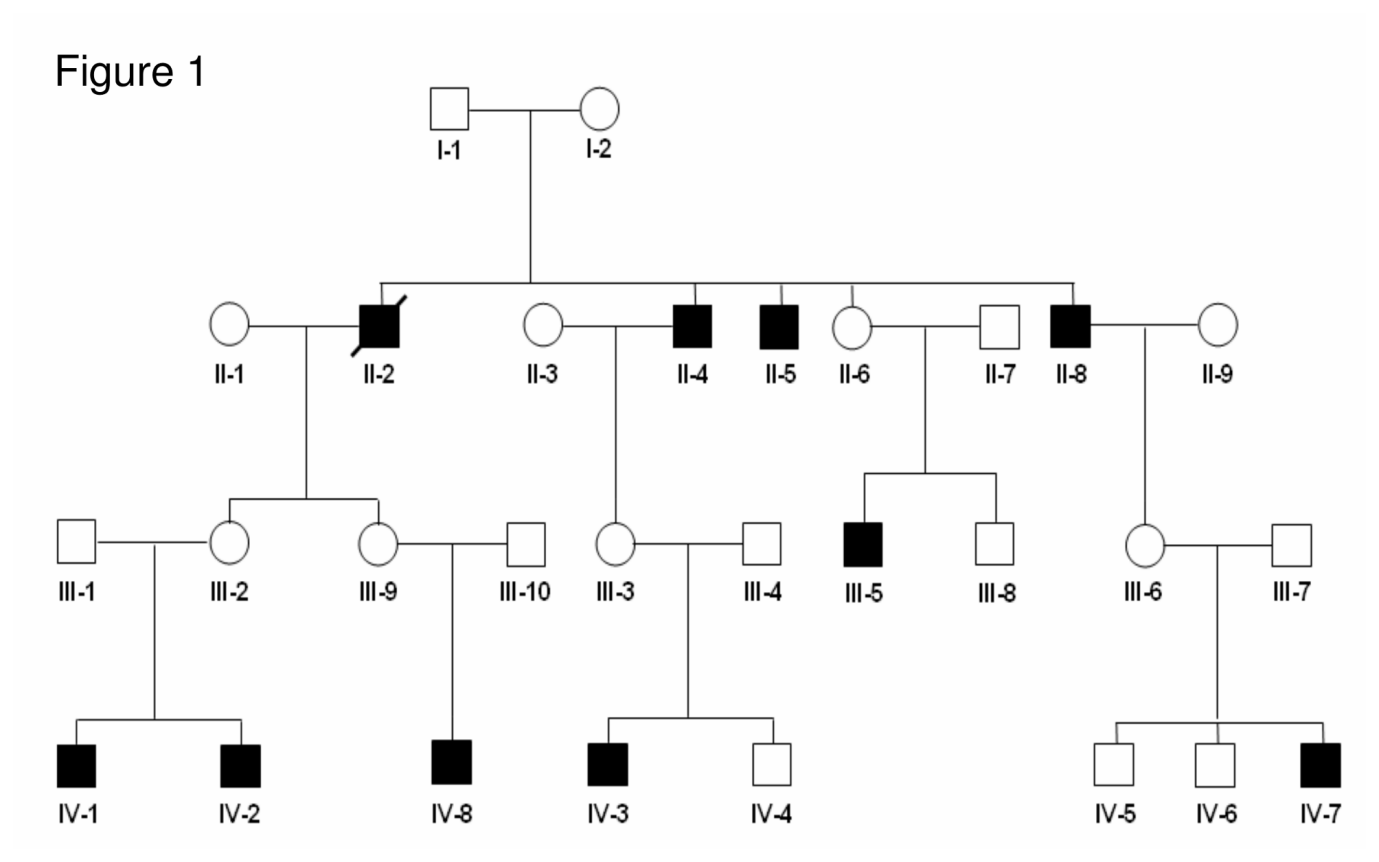
Pedigree of the family compatible with an x-linked recessive mode of inheritance. Four of the 10 affected males (IV-7, IV-8, III-5 and II-8) and two female carriers (III-6 and III-9) have been examined.

Written informed consent was obtained from all participating family members and in case of children from their parents or legal guardian(s) according to German law (Gendiagnostikgesetz) before conducting any genetic testing. Genetic testing was performed on a diagnostic basis in all affected males and female carriers after proper genetic counseling. All family members and parents in case of minors approved the anonymous use of their clinical and genetic data for clinical research in the setting of eye disease. The clinical research was conducted in accordance with the principles of the Declaration of Helsinki. This study was approved by the local ethics committee (University of Cologne, Kerpener Str. 62, 50937 Cologne, Germany, Az10-114, 3/6/2010 and Az13-189, 10/7/2013). Genomic DNA derived from 10 family members (II-4, II-6, II-8, III-5, III-6, III-7, III-8, III-9, IV-7, IV-8) (Figure 1) was isolated from venous blood samples using standard methods [17]. DNA derived from unrelated healthy controls was isolated from saliva using the SalivaGene Collection Module II (STRATEC Molecular GmbH, Berlin, Germany) according to the manufacturer’s protocol.

### Linkage analysis and whole X-exome sequencing

Samples from the indicated individuals were subjected to a whole genome scan, using the Affymetrix GeneChip Human Mapping 250K Nsp Array. Relationship errors were evaluated with the help of the program Graphical Relationship Representation [18]. The program PedCheck was applied to detect Mendelian errors [19]. Non-parametric linkage analysis was performed with MERLIN [20]. Parametric linkage and haplotype analysis were carried out using ALLEGRO [21], assuming recessive inheritance, full penetrance and a disease gene frequency of 0.0001. All data handling was performed using the graphical user interface ALOHOMORA [22]. Targeted next generation X chromosome sequencing was conducted using the Agilent SureSelect Human X Chromosome Kit and the Illumina Genome Analyzer IIx. Reads were aligned against the human reference genome (build hg19) using Illumina’s CASAVA software. SNPs and short Indels were called using samtools [23]. Indels were only considered for further analysis if present in the reads with frequency at least 10% and all variants present in dbSNP and the 1000 Genomes Project were filtered out. Finally, only variants lying within the previously identified linkage regions were considered for further inspection. For visual inspection of the coverage, wiggle files were created from the alignments using samtools and self-written scripts. They were further converted to bigwig files using the UCSC Genome Browser’s wigToBigWig [24] tool and uploaded to the UCSC Genome Browser. Coverage of every exon annotated in Ensembl Build 63 was computed and compared between samples using self-written scripts. The issue of privacy protection remains highly controversial and our ethics committee finds the deposition of deep sequencing data problematic as reidentification cannot be excluded. We are not allowed to deposit the data in a public repository at this time. However, data can be shared upon request.

### Mutational analysis

Mutations in the alternatively spliced open reading frame 15 variant (ORF15) of the retinitis pigmentosa G-coupled receptor (RPGR) gene were excluded by Sanger sequencing using the protocol described below. The exons 17 to 27 of the CACNA1F gene (NM_005183.2) including adjacent intronic sequences were amplified by PCR (20 ng of genomic DNA, 25 µl reaction mixture containing 10 pmol of each forward and reverse primers) using the Qiagen Multiplex PCR Kit (Qiagen, Hilden, Germany). PCR products were visualized on 1.5% agarose gels by ethidium bromide staining. For sequencing of the junction fragment, PCR products were digested with exonuclease I and shrimp alkaline phosphatase (Fermentas Life Sciences, Glen Burnie, MD) and sequenced using BigDye Terminator v.1.1 kit (Applied Biosystems, Darmstadt, Germany). Primers are listed in Table S1.

### RNA isolation and RT-PCR

RNA isolation was performed using the RNeasy Mini Kit (Qiagen, Hilden, Germany). RNA concentrations were determined using a NanoDrop ND-1000 spectrophotometer (Peqlab, Erlangen, Germany). Reverse transcription (RT) was performed using oligo dT primers and 1 μg of total RNA by applying the SuperScript First-Strand Synthesis System (Invitrogen, Karlsruhe, Germany). 2 µl of each 20 µl RT reaction was used for each RT-PCR. The following primer pair was used for the amplification of *CACNA1F* transcripts: CACNA1F_exon14_fwd: CTG TTC ACG GTG GAG ATG CTT, CACNA1F_exon27_rev: GCC GTG ACA CGT CTC CAT CT. Human Retina Marathon-Ready™ cDNA (Clontech, TAKARA Bio, Europe, Saint-Germain-en-Laye, France) was used as positive control. PCR conditions were as follows: 5 min initial denaturation (94°C), 40 cycles (94°C for 30 sec, 60°C for 30 sec, 72°C for 40 sec) and a final extension step for 5 min at 72°C. PCR products were visualized on 1.5% agarose gels by ethidium bromide staining.

### Clinical evaluation

The ophthalmologic examination comprised refraction, best corrected visual acuity, anterior segment examination, fundoscopy with dilated pupil, fundus photography and spectral domain optic coherence tomography (Spectralis HRA+OCT, Heidelberg Engineering GmbH, Heidelberg, Germany). Fundus autofluorescence imaging was performed in two patients (III-5, IV-7), fluorescence angiography and indocyanine green angiography (Spectralis HRA+OCT) in one patient (III-5). Colour vision was tested with Ishihara plates and/or Farnsworth Panel D-15 test in the school boy and the two adults. The orthoptic examination included measurement of binocular function with Bagolini striated glasses test, Titmus stereofly test and Lang test. Strabismus was examined by prism-cover test and by alternating cover test with prism at near and far fixation. The visual fields were tested monocularly with the Goldmann perimeter (Haag-Streit, Wedel, Germany) and/or Octopus perimeter (Haag-Streit, Wedel, Germany), dependent on the visual acuity and cooperation of the patient. Dark adaptation was tested with the Goldmann-Weekers adaptometer (Haag-Streit, Wesel, Germany) in one affected male. Clinical findings concerning affected members of the family who were not able to participate in an examination at our hospital were reviewed from chart data if available including age, gender, age at first onset of symptoms, chief complaint, persistence of clinical symptoms and visual acuity. Electrophysiologic examination was performed in four affected males and two female carriers. Full-field ERGs (RETIPort, Roland Consult, Germany) were recorded from both eyes with gold foil electrodes in accordance with International Society for Clinical Electrophysiology of Vision guidelines in both scotopic and photopic conditions [25]. In the youngest boy, a white-flash screening ERG was recorded using non-corneal skin electrodes.

## Results

We analyzed a large family of German origin (Figure 1) with 10 males out of three generations affected from an inherited progressive retinal disorder. The presence of solely male affected individuals suggested a X-chromosomal recessive trait. Four patients (II-8, III-5, IV-7 and IV-8) and two obligate female conductors (III-6 and III-9) underwent detailed ophthalmologic examination at the Department of Ophthalmology, University Hospital of Cologne, in 2011. Of those, two patients have been examined previously at the Department of Ophthalmology, Cologne (III-5 in 2006; IV-7 in 2006 and 2010). Additional past medical records were available for the patients II-4, II-5, III-5 and IV-7. The detailed phenotypic characteristics of the probands II-8, III-5, III-6, III-9, IV-7 and IV-8 are given in Table 1.

**Table 1:** Phenotypic characterization of the family members II-8, III-5, IV-7, IV-8, III-6 and III-9.

**proband**	**II-8 (affected)**	**III-5 (affected)**	**IV-7 (affected)**	**IV-8 (affected)**	**III-6**	**III-9**
**refraction at last examination**	OD -21.00-0.25/74°; OS -21.25sph	OD -23.00sph; OS -22.75-0.5/43°	OD -8.00-3.00/5°; OS -7.50-3.00/165°	OD -2.75-2.25/160°; OS -3.0-1.5/174°	OD/OS emmetropia	OD +1.00-0.25/106°; OS +1.25-1.00/48°
**age at last examination (y)**	72	5151	8	3	45	43
**best corrected visual acuity OD/OS (decimal scale)**	0.01/0.014	0.16/0.2	0.2/0.2	0.125/0.125	1.0/1.0	1.0/0.8
**nystagmus**	congenital	congenital	congenital	no	no	no
**orthoptic examination**	orthotropia	orthotropia	esotropia	intermittent exotropia	orthophoria	esophoria
**binocular vision**	none	Bagolini Striated Glasses Test	none	Lang-Test localized, Titmus Fly	Lang-Test	Lang-Test
**biomicroscopy**	Cataracta provecta	Cataracta incipiens	normal	normal	normal	normal
**fundoscopy**	myopic changes (including pigmentary abnormalities at the posterior pole)	myopic changes (including pigmentary abnormalities at the posterior pole)	discreet optic nerve atrophia, irregular pigmentation in the macular area	discreet optic nerve atrophia, irregular pigmentation in the macular area	normal	normal
**SD-OCT**	with artifacts (staphyloma, nystagmus)	with artifacts (staphyloma, nystagmus)	thin choroid, otherwise normal; fovea normal	OS: fovea normal, OD: not reliable (cooperation)	normal	normal
**visual fields**	Goldmann III 4e normal	Goldmann III 4e normal	Goldmann III 4e normal; 30.2 reduced sensitivity	not examined	30.2 normal	30.2 normal
**color vision**	severe red-green defects	moderate red-green defects	mild red-green defects	not examined	normal	normal
**dark adaptation**	not examined	not examined	elevated rod and cone thresholds (2 log units)	not examined	not examined	not examined
**scotopic ERG**	severely reduced, almost extinguished a- and b- wave	severely reduced, almost extinguished a- and b-wave	slightly reduced a- and b-wave, no “negative” ERG	screening ERG below noise level	low, but within normal ranges	low, but within normal ranges
**photopic ERG**	not recordable	not recordable	below noise level	screening ERG below noise level	normal	normal
**oscillatory potentials**	not recordable	not recordable	reduced	not examined	normal	normal
**30 Hz flicker ERG**	not recordable	not recordable	not recordable	not examined	normal	with artefacts

OD = right eye, OS = left eye; SD-OCT = Spectral domain optic coherence tomography; ERG = electroretinogram

The oldest patient (II-8, 72 years at examination) suffered from photophobia, decreased visual acuity, and severe myopia. Patient III-5 (51y at last examination) exhibited decreased visual acuity (OD (right eye) 0.16; OS (left eye) 0.2), colour vision defects and severe myopia (OD -23.00 D, OS -22.75 D). The patient reported the severity of symptoms to be progressive. Concordantly, according to the past medical records myopia was less pronounced (OD -16.25 D; OS -16.25 D) and visual acuity less reduced (OD 0.2; OS 0.3) at the patients’ age of 26 years. Patient IV-7 (8 years at last examination) suffered from reduced visual acuity and nystagmus, but did not report photophobia or nyctalopia. The parents of the youngest patient (IV-8, 3 years at examination) had not noticed any abnormalities besides intermittent exotropia, especially no nystagmus. All examined affected males showed mild to severe myopia (spherical equivalent -2.75 D to -23.00 D) with older patients (II-8, III-5) requiring higher corrections. Two patients showed astigmatism of more than 1.5 D. The visual acuity of the affected patients ranged from 0.05 (20/400) tested at 1 m distance to 0.2 (20/100) tested at 5 m distance. Three of the four patients showed nystagmus referred to be congenital (IV-7, III-5, II-8), two showed strabismus (IV-8, IV-7). Colour vision was defective in the school boy (IV-7) and severely reduced in the two adults (III-5, II-8). None of the patients reported difficulties with vision in dim conditions, although the dark adaptation showed elevated rod and cone thresholds in one patient examined. According to the past medical records, patient II-4 suffered from nystagmus, strabismus convergens alternans and high myopia at the age of 51 years, while nystagmus, decreased visual acuity and myopia was reported for patient II-5.

**Figure 2:**
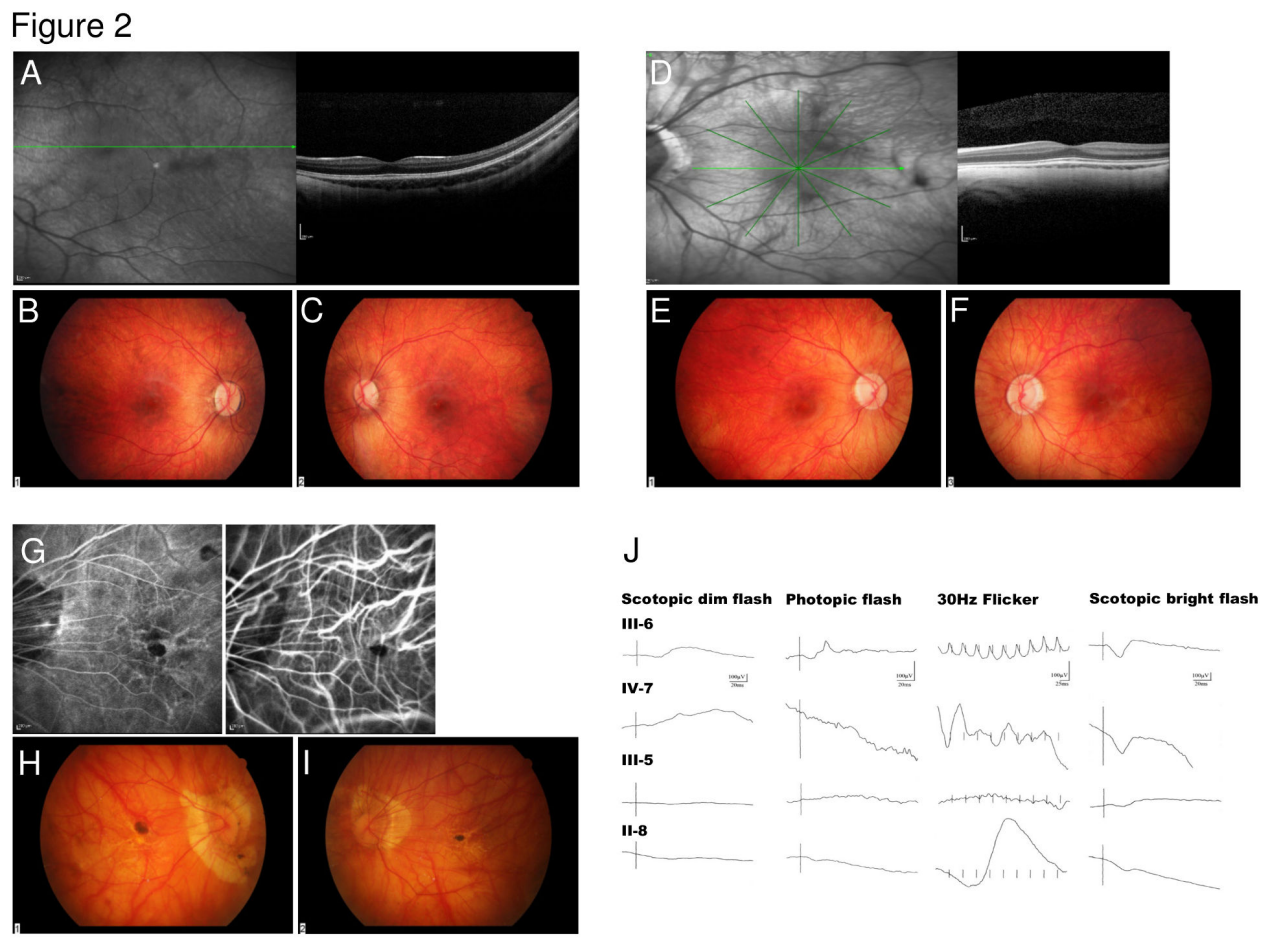
Ocular phenotypic characteristics of patient IV-7. Spectral domain optic coherence tomography (SD-OCT) of the left eye shows a normal foveal contour (A).Fundus photographies show discreet palor of the optic nerve (**B**) and irregular pigmentation in the macular area (**C**). Ocular phenotypic characteristics of patient IV-8: Spectral domain optic coherence tomography (SD-OCT) of the left eye shows a normal foveal contour (**D**). Fundus photographies show discreet palor of the optic nerve (**E**) and irregular pigmentation in the macular area especially in the right eye (**F**). Ocular phenotypic characteristics of patient III-5: Fundus fluorescence and indocyanine green angiography of the left eye show no leakage (**G**, **H**). Fundus photographies show myopic changes: posterior staphyloma, lacquer cracks, Fuchs’ spot of the macula and chorioretinal and parapapillary atrophy (**I**). Electrophysiologic examination (**J**): Full-field ERG shows normal scotopic and photopic answers in the female carrier (III-6). In the school boy (IV-7) ERG showed slightly reduced scotopic a- and b-waves with photopic answers below noise level. In the two adults (III-5, II-8), scotopic a- and b-waves were severely reduced with non-recordable photopic ERG.

Fundus photographies and OCT of the two younger affected males (IV-7, IV-8) are shown in Figure 2, A-F. Both boys showed discreet pallor of the optic nerve disc and discreet irregular pigmentation in the macular area. OCT examination revealed a normal foveal configuration and thinning of the choroids, which was more distinctive in the older child with higher myopia (IV-7). Fundus autofluorescence imaging was unremarkable in two affected family members. Fluorescence angiography revealed no leakage in one patient (III-5) with myopic fundus changes like posterior staphyloma, lacquer cracks, Fuchs’ spot of the macula and chorioretinal and parapapillary atrophy (Figure 2, G-I). Full-field ERG in the school boy (IV-7) showed slightly reduced scotopic a- and b-waves with photopic answers below noise level. In the two adults (III-5, II-8), scotopic a- and b-waves were severely reduced with non-recordable photopic ERG (Figure 2, J). The screening ERG in the youngest patient (IV-8) was below noise level. Both female carriers (III-6, III-9), aged 45 and 43 years at examination, respectively, were asymptomatic. One showed no refractive error, the other a mild hyperopic refraction and mild astigmatism. Visual acuity was 1.0 in each right eye, 0.8 and 1.0 in each left eye. The ophthalmologic findings, visual fields and colour vision were normal in both females. In the orthoptic examination, one of the female carriers showed an esophoria. Electrophysiologic examinations showed scotopic and photopic a- and b-waves within low normal ranges (Figure 2, J).

**Figure 3:**
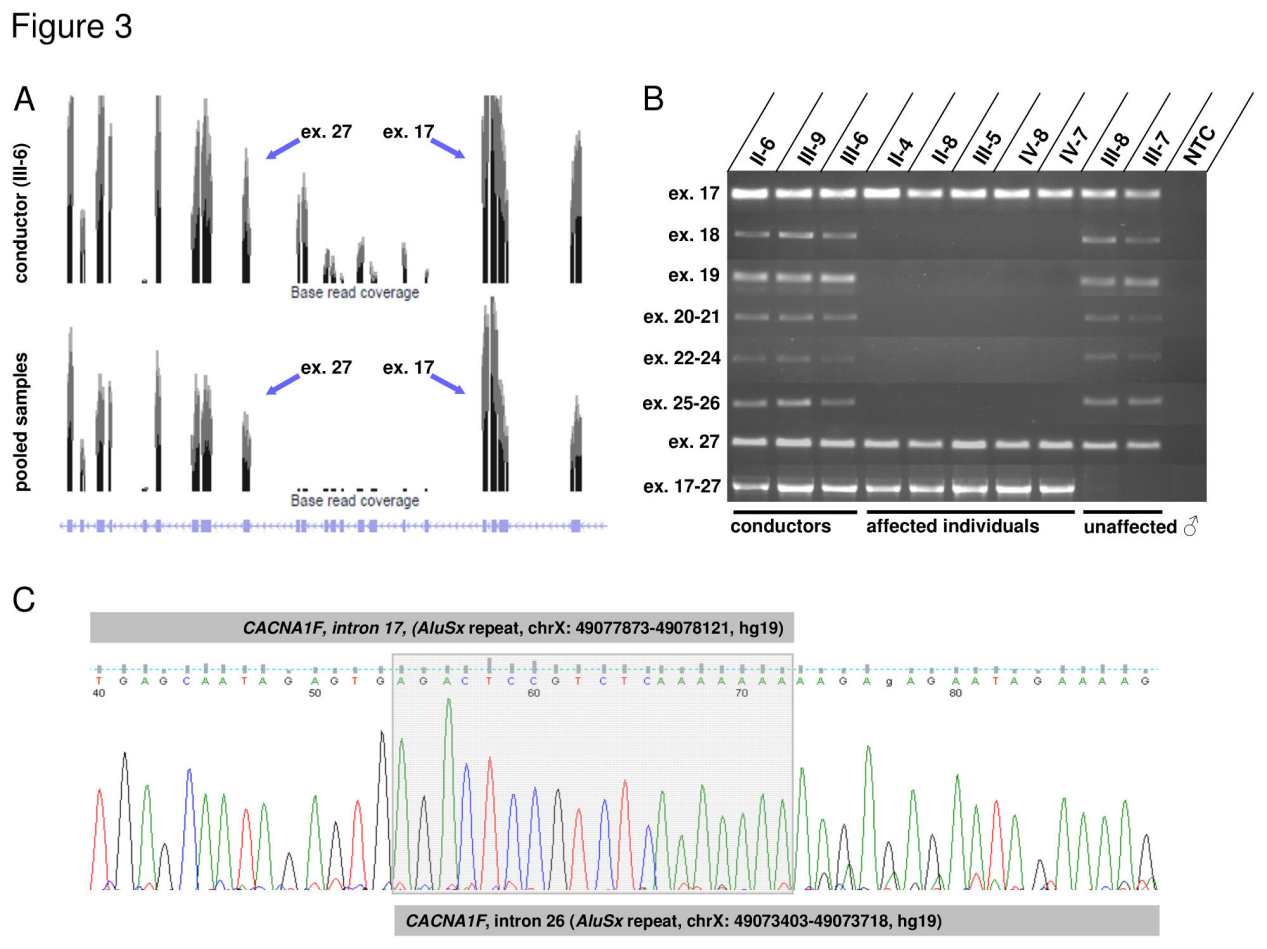
Graphical representation of *CACNA1F* NGS coverage (exons 14 to 35) for individual III-6 (conductor) versus pooled DNA samples derived from affected males. The genomic structure of *CACNA1F* (exons 14-35) is shown below. NGS coverage data suggest reduced signals of the *CACNA1F* exons 18-26 for individual III-6 while signals for those exons are absent in the pooled DNA sample (**A**). Analysis of genomic DNA shows junction fragment PCR-products only in patients and carriers but not in controls (**B**). Exons 18-26 were absent in all patients tested. NTC = no template control. Sequencing of the junction fragment product revealed breakpoints within two AluSx repeats located in intron 17 and 26 (**C**).

**Figure 4:**
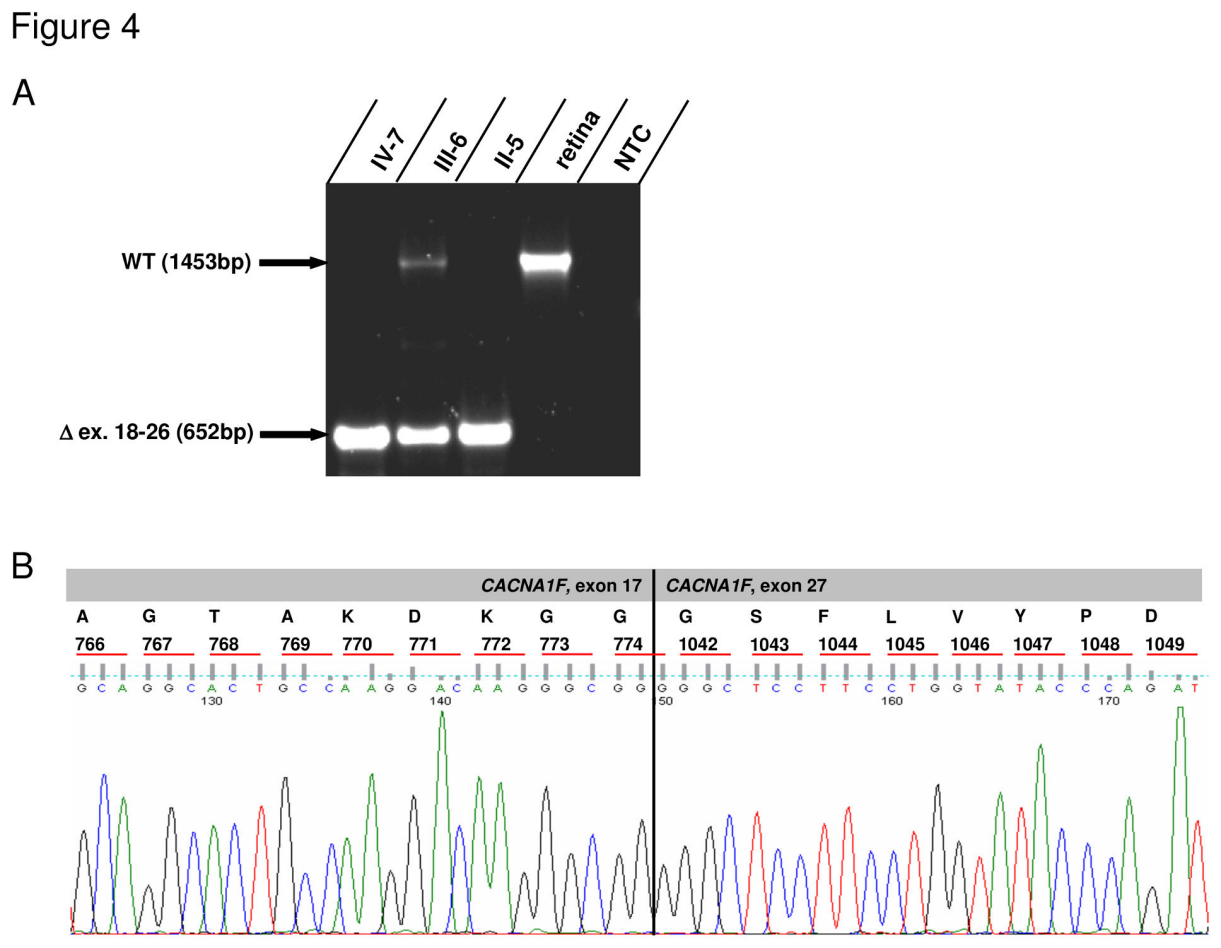
Analysis of cDNA derived from EBV-transformed lymphoblastoid cell lines (LCLs) of individuals IV-7 (affected), III-6 (female carrier) and II-5 (affected) shows expression of the truncated transcript in all samples while the WT-transcript was present only in the female carrier and the positive control (Human Retina Marathon-Ready™ cDNA). NTC = no template control.

The slowly progressive loss of visual acuity, moderate to high myopia, colour vision defects, affected cone and rod thresholds in dark adaptation, diminished cone-rod responses by full field ERG, irregular pigmentation in the macular area in the younger patients together with the mode of inheritance were suggestive for a X-linked cone-rod-dystrophy, CORDX [11,14]. However, the occurrence of nystagmus and astigmatism present in the family described above is considered atypical in CORDX3 and rare in CORDX1/2 cases [11,15]. By initial Sanger sequencing analysis (patient IV-7), we excluded a causative mutation within *open reading frame 15 variant* (*ORF15*) of the *RPGR* gene commonly associated with CORDX1 (data not shown). We next performed a genome-wide linkage analysis using 250 k SNP arrays and identified linkage to three X-chromosomal regions flanked by the markers rs5906215 and rs5906873 (Xp11.3-p11.23), rs6521410 and rs5919577 (Xp11.21-q12), as well as rs4892539 and rs12394799 (Xq13.3-q21.1). Maximum LOD scores of 2.4 were obtained for each region which was the maximum expected from simulation analysis (data not shown). The linked intervals neither contain the CORDX1-causing *RPGR* gene, nor overlap with the previously identified CORDX2 region. However, the interval located on Xp11.3-p11.23 contains the *CACNA1F* gene shown to cause CORDX3 [15]. Subsequently, we employed a novel next generation sequencing (NGS) approach in order to identify the causative alteration. Equal amounts of DNA derived from four patients (II-4, II-8, III-5 and IV-7) were pooled and, in addition to DNA derived from the obligate female conductor III-6, subjected to NGS. By focusing on exonic sequences and invariant consensus splice sites, NGS did not reveal any alterations within the linked regions, which were not present in SNP databases (1000 Genomes, Exome Variant Server; data not shown). Strikingly, the analysis of the NGS coverage data of each *CACNA1F* exon revealed a reduced coverage of the exons 18 to 26 (compared with flanking exons) in the female conductor III-6, and no coverage of these exons in the pooled patient’s DNA sample, suggesting a gross deletion (Figure 3). To confirm this finding, exons 17 to 27 were PCR-amplified using DNA derived from 3 female conductors (II-6, III-9, III-6), 5 affected individuals (II-4, II-8, III-5, IV-8, IV-7) and 2 unaffected male family members (III-7, III-8). While the exons 17 and 27 were present in all individuals, exons 18 to 26 were not amplifiable in patients’ DNA (Figure 3). To unambiguously confirm a gross genomic deletion, we were able to amplify a junction fragment in patients and female conductors, but not in unaffected male family members (Figure 3). A junction fragment was not observed using DNA derived from 50 male control individuals (data not shown). Sequencing of the junction fragment identified a gross deletion of parts of the introns 17, 26 and interjacent sequences. RepeatMasker analysis revealed that both introns harbour one Alu repeat of the AluSx family, which share a 70% identity (*blastn* analysis). The observed deletion is flanked by these AluSx repeat sequences on both sides, suggesting that its occurrence was the product of an Alu-Alu repeat-mediated nonhomologous recombination (Figure 3). The genomic breakpoint is located within a 19bp region which is identical between both flanking AluSx repeats (Figure 3). In order to analyse the consequences of the genomic deletion on transcript level, we generated EBV-transformed lymphoblastoid cell lines (LCL) using blood samples from the obligate female conductor III-6 and two patients (II-5, IV-7). Following isolation of total RNA we were able to detect the wild-type *CACNA1F* transcripts by RT-PCR in cells derived from the female carrier III-6, but not in those derived from the patients II-5 and IV-7 (Figure 4). As expected, sequencing of the truncated transcripts derived from patients II-5 and IV-7 revealed an aberrant junction of exon 17 to exon 27, representing an in-frame deletion and loss of 267 amino acids (residues 775-1041) (Figure 4).

## Discussion

The *CACNA1F* gene encodes the poreforming subunit of a voltage dependent L-type calcium channel, the Ca_v_1.4 channel. Staining of the human retina with a Ca_v_1.4 specific antibody revealed expression in the outer plexiform layer, inner nuclear layer, inner plexiform layer and nerve fibre layer [26]. Ca_v_1.4 channels were shown to be expressed in both rod and cone terminals and to mediate neurotransmitter secretion at the ribbon synapses of the retinal photoreceptors [27-29], thus playing an important role in signal transmission from photoreceptors to second order retinal neurons. Pathogenic alterations of the *CACNA1F* gene are mostly associated with incomplete X-linked congenital stationary night blindness type 2A (CSNB2A, MIM 300071), a non-progressive retinal disorder. To date, more than 60 different *CACNA1F* mutations have been reported to cause CSNB2A according to the HGMD database. A fair number of these mutations have been functionally characterized and apart from complete loss of function or decreased channel expression some displayed changes in the gating behaviour, while in others no biophysical effect could be observed. Clinical features of CSNB2A include nyctalopia, decreased visual acuity, myopia in most cases, nystagmus and strabismus, with essentially normal fundus except for myopic changes [1,16,30-32]. Affected males typically show a Schubert Bornschein ERG with recordable but reduced scotopic b-waves and reduced photopic b-waves [33,34] demonstrating affection of rod and cone signalling to second order neurons [33,35-37]. However, a few *CACNA1F* alterations have been associated with CSNB2-related but distinctive phenotypes such as Åland Island eye disease (ÅIED) [38], X-linked cone-rod dystrophy (CORDX3) [15], or a retinal disorder associated with intellectual disability, respectively [39,40].

While CSNB2A and the rare allelic phenotypes CORDX3 and ÅIED have many clinical features in common, the phenotype observed in this study is most precisely described as CORDX3-like. Three of the four patients of the family described in this study show colour vision defects, which is not typical for CSNB2A (colour vision is severely impaired in the patients II-8 and III-5). The two younger patients showed irregular pigmentation in the macular area, whereas in CSNB2A the fundus is essentially normal except for myopic changes [32,33]. Whereas in adult patients myopic changes can include macular pigmentary abnormalities, such changes would not be expected in the young age and mild respectively moderate myopia of the two boys of our family [41,42]. For the adult patients of our cohort it is difficult to decide whether the macular changes result from early pigmentary abnormalities due to the retinopathy or from myopic changes. The characteristic electroretinogram in CSNB2A shows reduced scotopic b-wave amplitudes in response to bright flashes after dark adaptation, resulting in a negative wave [33,34]. Whereas residual scotopic b-waves and photopic b-waves are recordable in CSNB2A, the rod and cone function tested by full field ERG was nearly extinguished in two of our cases. These findings and the progressive nature of the disease differentiate the phenotype described here from CSNB2A. The same holds true for ÅIED, which is clinically characterized by fundus hypopigmentation, decreased visual acuity due to foveal hypoplasia without evidence of chiasmal misrouting, iris trans-illumination defects, nystagmus, astigmatism, protan colour vision defect, progressive myopia and defective dark adaptation [38,43,44]. None of our patients examined show iris trans-illumination defects or fundus albinism and there were no signs for foveal hypoplasia in the fundus examination or OCT. According to the clinical features in the family described, a CORDX3-like phenotype appears to be the most accurate designation, though congenital nystagmus and astigmatism have not been associated with CORDX3 so far [11,15].

To date, a large phenotypic variability has been described for pathogenic *CACNA1F* mutations. For example, Hope and colleagues described a large New Zealand family with a novel *CACNA1F* mutation. Distinctive features present in this family include the association with intellectual disability in affected males and, most strikingly, electrophysiological and clinical abnormalities in female heterozygotes (nystagmus, decreased visual acuity, frequently high myopia) [39]. Functional characterization of the *CACNA1F* mutation found in this family suggests a gain-of-function mechanism involving increased Ca_v_1.4 channel activity, which is likely to cause the unusual phenotype observed [40]. In contrast, there is ample evidence that the loss of *CACNA1F* gene function causes CSNB2A. For example, nonsense mutations resulting in a protein truncation already within the first third of the 1977 amino acid CACNA1F protein such as Arg50Term, Arg82Term, Glu278Term, Gln439Term, or Arg625Term associate with CSNB2A [45-47] and concordantly, *Cacna1f* null-mice show a CSNB2A-like phenotype [48]. The CACNA1F protein consists of four homologous domains (I-IV) and each domain is comprised of six transmembrane helices [49]. The gross in-frame deletion identified in our family results in the loss of 4 transmembrane helices within the homologous domain III and therefore most likely affects function via altered receptor topology or protein targeting. As mutant *CACNA1F* mRNA could be detected in lymphoblastoid cell lines we assume that protein expression may not be affected *per se*. However, we have neither performed western blot analysis to document stability of the mutant protein nor electrophysiology studies to asses channel function in detail. Since pathogenic mutations could be identified in all homologous domains, all four seem to be necessary for proper channel function.

In summary, the variable clinical features observed in CSNB2A and its allelic disorders ÅIED and CORDX3 are not explainable by differing consequences of different *CACNA1F* mutations on protein function. Boycott and co-workers reported the clinical findings of a group of 66 male patients with CSNB2A of Mennonite ancestry that share the same 3166-3167insC mutation. At least one of the major features of CSNB2A was absent in 72% of the patients, and all of the examined features varied widely both between and within families [31]. In the absence of a clear genotype-phenotype correlation, it appears likely that additional yet unknown *CACNA1F*-independent disease modifying factors exist. This notion is supported by the recent finding that CSNB2A and ÅIED phenotypes coexist in a family with a *CACNA1F* missense mutation [50]. Hence, the molecular bases of the allelic phenotypes remain elusive and require further investigation. However, our data independently confirm *CACNA1F* as the causative gene for CORDX3-like phenotypes and detailed clinical characterization of the family expands the knowledge about the phenotypic spectrum of *CACNA1F* mutations.

## Supporting Information

Table S1 **Oligonucleotides used for the amplification of *CACNA1F* exons 17 to 27 and junction fragments (gDNA, cDNA).**
(DOC)
