# Supplementary material for: A Novel Large In-Frame Deletion within the CACNA1F Gene Associates with a Cone-Rod Dystrophy 3-Like Phenotype
Source: PLoS One. 2013 Oct 4;8(10):e76414. doi: 10.1371/journal.pone.0076414 (PMC3790679; doi:10.1371/journal.pone.0076414)
Supplement: Table S1 — Oligonucleotides used for the amplification of CACNA1F exons 17 to 27 and junction fragments (gDNA, cDNA). (DOC) [file pone.0076414.s001.doc]

| **amplicon** | **forward Primer** | **reverse Primer** | **amplicon length** |
| --- | --- | --- | --- |
| **oligonucleotides used for the amplification of CACNA1F exons 17 to 27** |  |  |  |
| CACNA1F exon 17 | CTG GAT GAT GGT GGA GTC G | TAG AGG CAT CTC TGG TGG TG | 264bp |
| CACNA1F exon 18 | GCT GGA ATG GAG TGA GGA AG | GGC AGA GGA TGA AGG TTA AAG | 783bp |
| CACNA1F exon 19 | TCT TTA ACC TTC ATC CTC TGC C | TGG AAA AGG CTG ATC TCT GG | 278bp |
| CACNA1F exon 20-21 | AAT GGG AAG AAT TGA CCA CG | TTA TCT CCA CCC TGA CCC AG | 647bp |
| CACNA1F exon 22-24 | ATC TTT CTG ATG GCC AAG CC | CAT CCG TGC AAA TGG TCA G | 667bp |
| CACNA1F exon 25-26 | CTC AGC TGC ATC TAT GAC CAG | TAT CTT TGG GCC TTG GTG AG | 424bp |
| CACNA1F exon 27 | TGC TCC ACC ACT ATG TCC TG | ACT CCC ACC CAT TCC CAG | 336bp |
| **oligonucleotides used for the amplification of junction fragments (gDNA, cDNA)** |  |  |  |
| CACNA1F gDNA | CTG GAT GAT GGT GGA GTC G | ACT CCC ACC CAT TCC CAG | ~6.4 Kb (wt), ~1.8Kb (del) |
| CACNA1F cDNA | CTG TTC ACG GTG GAG ATG CTT | GCC GTG ACA CGT CTC CAT CT | 1453bp (wt), 652 bp (del) |
